# Worse than the Insult: The Role of Macrophages and Lymphocytes in Organic Dust-Induced Chronic Bronchitis

**DOI:** 10.3390/cells15212013

**Published:** 2026-11-05

**Authors:** Jenny Pakhrin Rana, Joseph L. Graves, Radiah C. Minor, Jenora T. Waterman

**Affiliations:** 1 Department of Biology, North Carolina Agricultural and Technical State University, Greensboro, NC 27411, USA; jpakhrinrana@aggies.ncat.edu (J.P.R.); gravesjl@ncat.edu (J.L.G.J.); 2 Department of Animal Sciences, North Carolina Agricultural and Technical State University, Greensboro, NC 27411, USA; rcminor@ncat.edu

**Keywords:** organic dust, airway inflammation, chronic bronchitis, macrophages, lymphocytes, cytokines, signaling pathways

## Abstract

**Highlights:**

**What are the main findings?**

- Repetitive exposure to organic dust, or its components including LPS, may promote sustained activation of macrophages and lymphocytes.
- Unresolved inflammation associated with repetitive organic dust exposure drives the structural changes in the airways that may lead to chronic bronchitis.

**What is the implication of the main finding?**

- The review provides a mechanistic explanation for organic dust-associated chronic bronchitis.
- Defining the efficacy of cellular and molecular targeting agents is essential for prevention or relief of prolonged airway inflammation associated with organic dust exposure.
- Chronic exposure to organic dust in swine production facilities results in activation of macrophages and lymphocytes in lungs via multiple signaling pathways including NF-κB, MAPK, and PKC pathways.
- Activation of these signaling pathways results in hypersecretion of pro- and anti-inflammatory cytokines, release of reactive oxygen and nitrogen species, and changes in airway pathophysiology.
- Understanding the role of alveolar macrophages and lymphocytes and the involved signaling pathways provides insight into the mechanism of organic dust-induced chronic bronchitis.

**Abstract:**

In livestock production settings, repetitive exposure to organic dust (OD) is a risk factor for chronic bronchitis (CB). Macrophages and lymphocytes cooperate to defend the lungs against inhaled dust, but when chronically activated, their actions contribute to chronic inflammation, oxidative stress, and tissue remodeling. Despite the strong body of literature examining the effects of OD exposure on leukocytes and lungs, the contribution of macrophages and lymphocytes to organic dust-associated chronic bronchitis is not fully understood. This review examines the role of macrophages and lymphocytes in organic dust-associated chronic bronchitis. To identify the body of literature addressing this goal, we conducted Boolean searches with keywords (e.g., bronchitis, leukocytes, organic dust) across four major databases: PubMed, Science Direct, Scopus, and Web of Science. From a total of 25,488 hits from all time, 18 research articles (from 2015 to 2025) provided contextual connection for explaining organic dust-associated chronic bronchitis. The results showed continuous or repetitive exposure to OD or its components (e.g., lipopolysaccharide) causes prolonged activation of macrophages and lymphocytes, which leads to overexpression of pro- and anti-inflammatory cytokines and chemokines via Toll-like receptor (TLR)4, TLR2, nuclear factor kappa B, protein kinase C, Extracellular Signal-Regulated Kinase (ERK), and Mitogen-Activated Protein Kinase (MAPK) pathways. Unresolved lung inflammation drives pathologic changes including mucus hypersecretion, airway remodeling, and onset of chronic bronchitis symptoms. Addition of micronutrients or other anti-inflammatory treatment helps to mitigate OD-induced lung inflammation. Defining the cellular and molecular contributions to organic dust-associated chronic bronchitis is essential for the development of targeted therapies that prevent or relieve prolonged airway inflammation.

## 1. Introduction

Disease can be caused by seven primary ultimate causes: genetics, environment, by-products of defense, lack of maintenance, disruption of homeostasis, stochastic events during development, and fitness conflicts between relatives [1]. Any specific disease may result from multiple ultimate causes. Chronic bronchitis (CB) is a disease that is caused by a combination of genetics, environment, and as a by-product of defense, specifically inflammation. CB, a long-term inflammatory (by-product of defense) airway disease, arises from repetitive exposure to noxious substances (environment). The prolonged, unresolved inflammation can result in decreased lung function, respiratory complications, and increased morbidity in severe cases. Genetics may play a role as some individuals will be more susceptible to immune hypersensitivity, and its impacts are also age-specific, with aging (senescent) individuals much more likely to display adverse disease than younger (non-senescent) individuals. In addition to chronic bronchitis, other respiratory complications observed in the agricultural farming environment are hypersensitivity pneumonitis, asthma, allergic and non-allergic rhinitis, and chronic cough with phlegm [2,3,4]. These diseases share the same ultimate causality with chronic bronchitis. The development of chronic bronchitis in the agricultural farming environment, especially in individuals exposed to livestock production facilities, is associated with repetitive exposure to organic dust (OD) present in those facilities [2,5].

Over the past several decades, there has been increasing evidence to suggest that exposure to organic dust and livestock production settings including swine buildings can contribute to the onset of pulmonary symptoms and disease including increase in bronchial responsiveness, decrease in forced expiratory volume, chronic cough, decline in lung function, and chronic bronchitis in humans [6,7,8] Livestock and dairy farmers developed diagnostic markers indicating possible bronchial obstruction such as chronic cough, chronic bronchitis, wheeze, and dyspnea with reduced FEV1 [9,10]. Similarly, poultry farmers, who worked for long periods (>2 h daily), developed similar symptoms, which were detected upon pulmonary function test, with a risk factor being exposure to organic dust present in the building [11]. Upon measurement, principal contaminants found in the farms were organic dust and endotoxin, where both dust and endotoxin were associated with COPD in a dose-dependent manner, especially in swine, cattle, and poultry farmers [11,12].

The United States (U.S.) is the third-largest global producer of pork, and the hog production market has increased by 40% over the last fifty years; harvesting over 120 million head in 2021 in the U.S. alone (with Iowa, North Carolina, and Minnesota being the three largest producers, respectively) [13]. In 2021, 67,000 farms averaging 1,080 hogs per farm were reported in the U.S. [13]. Livestock farmers are at greater risk of developing respiratory complications, especially the swine farmers, due to high demand and an increased number of pigs, large-scale production, and an increase in working hours in the confined building facilities for 8 h per day [14]. As a result, swine barn workers suffer from respiratory health issues such as impaired airflow, and lung inflammation due to inhalation of barn air containing pollutants, including OD [14]. Organic dust, present in livestock production facilities, consists of particulate matter (PM) ranging from inhalable (PM_10_) to respirable (PM_2.5_) dust and PM of organic origins such as bacteria, viruses, fungi, fecal matter, pollen, etc. [15,16]. In acute exposures, OD caused immediate changes, including airway damage, prior to returning to normal function after the causative agent is removed/cleared. Whereas repetitive or chronic inhalation of OD can result in permanent loss of cell function and disease [15,16,17,18]. For instance, studies of swine farmers reported that workers spent an average of 6.6 ± 1.8 h per day in production buildings and worked at those sites for 10.5 ± 7.5 years and thus are at increased risk of developing increased airway responsiveness, airflow obstruction, chronic cough, and phlegm compared to shorter daily contacts, including a higher prevalence of CB (21.94% vs. 13.25%) in those swine farmers [19,20,21].

Chronic bronchitis is defined by persistent cough and sputum production lasting at least 3 months for 2 consecutive years, and is one of two common clinical phenomena of chronic obstructive pulmonary disease (COPD) [22]. In addition to persistent productive cough, CB is characterized by mucus hypersecretion, chronic inflammation, obstruction of small airways, airway remodeling, alteration of airway surface tension, decreased lung function, shortness of breath, fatigue, and wheezing in severe cases [23,24]. Unlike emphysema, one of the two main subtypes of COPD, CB can occur independently in cases where affected individuals have a normal breathing test [22]. The prevalence of CB is 14–74% of all patients who have developed COPD [24]. In a study conducted from 2007 to 2012, among 213 million ever-employed adults, 52% of adults employed in industries considered to have workplace dust, vapors, or fumes exposure associated with CB and emphysema, whereas, people working in farming, fishing, and forestry occupations were one of the occupations to have higher prevalence of developing CB and emphysema [25]. The primary cause of CB is cigarette smoking; however, working within environments with a higher risk of exposure to dust, vapors, and fumes also leads to CB upon long-term exposure at a rate of 4.6% in about 213 million ever-employed adults (crude prevalence and with the rate of 2.3% in age-related prevalence) [25,26]. Among farming occupations, prevalence of CB is higher in swine producers compared to grain farmers [27]. Composition of OD could be responsible for this outcome. Exposure assessment of swine farms recorded endotoxin, hydrogen sulfide, bacteria, 1-3-β-glucans, ammonia, mites, fungi, and other dust from higher to lower level (measured from 1992 to 1996 during all seasons on swine farms) [6]. Moreover, the livestock air resistome profiles and the collection of airborne antibiotic resistance genes, are also high in swine farms [28]. Development of CB is associated with exposure to OD, fungal spores, bacteria, and endotoxin in the swine production [6].

**Figure 1:**
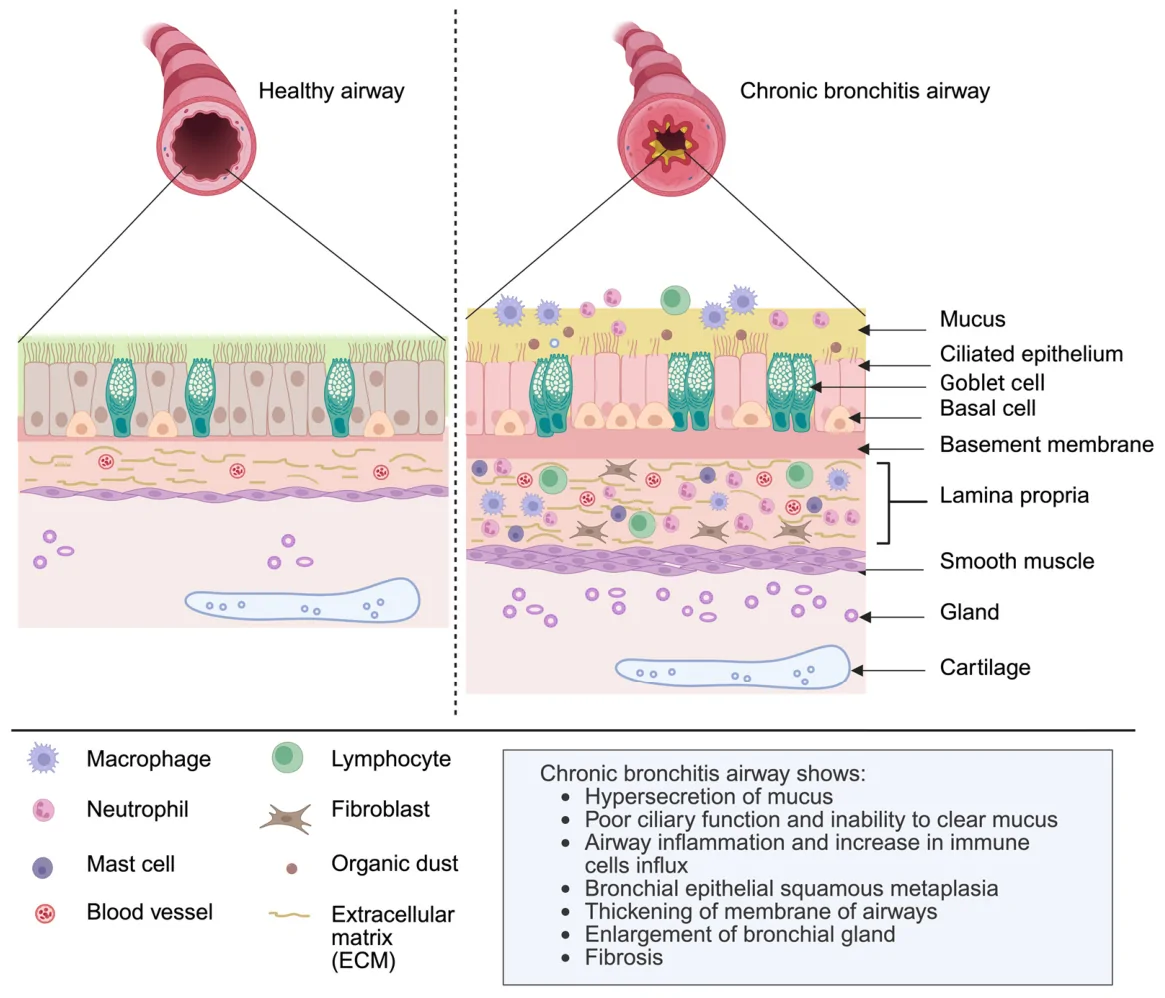
Healthy and chronic bronchitis airways (created with https://BioRender.com).

Acute OD exposure increases the total cells influx, whereas, chronic inhalation of OD increases inflammatory cells influx such as increase in neutrophils, macrophages, and lymphocytes, which is accompanied by increase in lymphoid aggregates and associated pro- and anti-inflammatory cytokines and chemokines [29,30]. These changes are compounded by pathologic changes in the lungs including increased size of the alveolar and bronchoalveolar compartment [22,31]. Moreover, the continuous influx of neutrophils results in increase in necrotic neutrophils that cannot be cleared by macrophages, thus resulting in increase of intracellular contents, elastase, and reactive oxygen species (ROS) in the sputum [22]. These necrotic neutrophils and their contents further increase the oxidative stress that perpetuate the cycle of inflammation followed by mucus overproduction and mucus hypersecretion by mucus glands and goblet cell degranulation [22]. Persistent airway inflammation and the inability to clear the mucus limits the airflow, increases airway wall thickness, fibrosis and smooth muscle hypertrophy leading to CB [22,32]. The change in pathophysiology during CB is shown in Figure 1. Besides neutrophilia, macrophages and lymphocytes play major roles in orchestrating inflammatory response in OD-induced CB [33]. For example, former swine farmers who were exposed to OD for 3 h in swine farm showed reduction in monocyte CD14 in blood and decrease in expression of alveolar macrophages (AMs) HLA-DR compared to baseline, correlating with inhalable LPS (r = −0.579, *p* = 0.048, n = 12) [34]. In contrast, CB patients or healthy subjects exposed to OD showed increased levels of monocytes, alveolar macrophages, and lymphocytes populations in serum, sputum, and bronchoalveolar lavage fluid (BALF) and decrease in forced expiratory volume (FEV1) [33,35,36,37,38,39]. In addition, proinflammatory cytokines related to macrophages and lymphocytes are also higher. Chronic bronchitis patients, swine farm workers, or healthy subjects exposed to swine barn OD with or without prolonged cigarette smoking showed elevated influx of macrophages, neutrophils, eosinophils, mast cells, CD8^+^ T lymphocytes, and other pro-inflammatory cytokines such as tumor necrosis alpha (TNF-α), interleukin (IL)-6, IL-2, and IL-8 [40]. The difference in macrophage activation upon swine dust exposure to former swine farmers and healthy individuals could be explained by the chronic activation and adaption response of macrophages [34].

Alveolar resident and exudate macrophages initiate and maintain pulmonary inflammation, defend against inhaled antigens, and maintain tissue homeostasis by assisting with repair and resolution of acute inflammation [41,42,43]. In lungs, macrophages reside in alveoli (alveolar macrophages) and mononuclear macrophages enter into lung tissue from the bloodstream in a chemokine receptor (CCR2)-dependent pathway, which are differentiated into interstitial macrophages and increased during pulmonary fibrosis [41,44]. Macrophages recognize OD or its components via toll-like receptor (TLR) pathway. Toll-like receptor 4 (TLR4) recognize Gram-negative bacterial infections via bacterial component, lipopolysaccharide (LPS), which is one of the OD components [45,46]. TLR4 activates transcription factors nuclear factor kappa-B (NF-κB), activation protein-1 (AP1), and cAMP-response element-binding protein (CREB), which further upregulates downstream pro-inflammatory cytokines [45,46]. In addition to TLR4, TLR2 (a pattern recognition receptor for Gram-positive cell wall products) also helps in regulating the inflammation induced by OD, where knockout of TLR2 pathway reduced the total cellular influx and TNF-α, IL-6, and Chemokine (C-X-C motif) ligand (CXCL)1 expression [47]. Furthermore, TLR4 299/399 polymorphism and TLR2-16933T/A polymorphism have been shown to be associated with decrease in airway hyperresponsiveness in response to endotoxin exposure and greater lung function than wild-type genotypes, respectively, suggesting their importance in mediating OD-induced airway responses [39,48].

After recognition of inhaled antigens, alveolar macrophages clear pathogens via phagocytosis and respiratory burst, which involves the generation of ROS [49]. In this process, ROS activate Nicotinamide adenine dinucleotide phosphate (NADPH) oxidase, which leads to enhanced production of ROS, including superoxide, hydroxyl radical, and hydrogen peroxide, which neutralize pathogens by inducing irreversible oxidative stress [50]. The generation of ROS occurs via several pathways such as nuclear factor kappa beta (NF-κB), activation protein-1 (AP-1), mitogen-activating protein kinase (MAPK), and phosphatidyl inositol-3 kinase (PI3K) pathway [49]. In mitochondria, the majority of ROS are produced due to continuous metabolism of oxygen, and similarly, NADPH oxidase also contributes to ROS production [51]. Receptors that bind to ROS, such as toll-like receptors (TLRs), nuclear oligomerization domain (NOD)-like receptors (NLRs), and other pattern recognition receptors (PRRs), depend on mitochondrial ROS signaling and activation to kill bacteria [52]. Activation of these pathogen recognition receptors (PRRs) by mitochondrial ROS leads to formation of inflammasomes that activate caspase-1, and cause proteolytic cleavage, maturation of proinflammatory cytokines such as IL-1β, and induce inflammation [52,53]. Activation of PRRs by pathogen-associated molecular patterns (PAMPs) and danger-associated molecular patterns (DAMPs) trigger downstream signaling to NOD-like receptor protein 3 (NLRP3), which activates NLRP3 inflammasome, a protein complex involved in the pathogenesis of inflammatory diseases [52,54]. PAMPs, including those present in organic dust such as LPS and peptidoglycan (PGN), are conserved bacterial components that trigger inflammation [55]. Macrophages (THP1) exposed to OD extract (ODE) increase mitochondrial DNA (mtDNA) leakage into the cytosol [55]. The leakage of mtDNA with ROS into the cytosol initiates the inflammatory cascade. Activated macrophages in association with epithelial cells release pro-inflammatory cytokines and mucin glycoproteins, especially mucin (MUC)5B, which are elevated in COPD and CB patients [33,56]. The changes in airway pathology and persistent mucus secretion lead to chronic cough and airway obstruction observed in COPD. On the contrary, macrophages re-exposed to high dose of ODE (1%), LPS, and PGN showed decrease in expression of monocyte-derived macrophages surface marker, cytokines, and phagocytosis (except LPS), signifying that OD and its components may impair macrophage phenotypes and functions [57]. Impaired phagocytosis by alveolar macrophages was observed in COPD and is associated with exacerbations caused by *Haemophilus* antigens [57,58]. Thus, the discrepancy in the function of macrophages shows that there is still a gap in understanding the macrophage response to chronic exposure to OD and its components.

Lymphocytes predominance with macrophages and other immune cells is a key signature of chronic inflammation in airways and lungs [59]. Organic dust stimulated macrophages to activate natural killer cells and T-lymphocytes via IL-22 pathway [60]. Lymphocytes influx is related to a key signature of CB, i.e., airflow limitation, because number of T-lymphocytes is inversely correlated with forced expiratory volume 1 (FEV1) in humans [61]. CB patients with airflow limitation also showed increase in smooth muscle area indicating the airway remodeling, which drives the pathogenesis of CB [62]. Healthy subjects showed increase in lymphocytes influx after three hours of exposure to OD in swine confinement building [35,36]. Similarly, the repetitive exposure to OD increases CD3^+^CD4^+^ T cells and CD3^+^CD8^+^ T cells in human and animals [60,63]. A week-long recovery also did not reduce the CD3^+^CD4^+^ T cells influx after OD exposure [2]. Other T-cells such as T-helper (TH)-17 and TH1 cells also increased due to OD exposure [2]. Whole blood exposed to OD showed a dose-dependent enhanced expression of activation markers CD69, CD25, and Human Leukocyte Antigen-DR (HLA-DR) [64]. Observance of these T-lymphocytes activation markers CD69, CD25, and HLA-DR represents early, middle, and late activation markers, respectively, present on CD3-lymphocyte [60,65]. Another subtype of lymphocytes, B-lymphocyte, helps to protect against microbial infection; however, higher levels of B cells are linked to the severity of chronic respiratory diseases, such as COPD, due to autoantibody response making its function in the lungs controversial [63]. In COPD patients, B cells, especially memory B cells (CD20^+^, CD27^+^) are higher in serum which underscore the role of B cells in “memory” and priming the system for rapid inflammation. Knockout of B cells resulted in increase in IL-17 and hyaluronan in wildtype (WT) and B-cell knockout animals compared to PBS control, with decrease in TNF-α, IL-6, CXCL1, and CXCL2 compared to WT mice, suggesting the role of B-cells in OD-induced inflammation [66].

Organic dust exposure, along with neutrophil and total cells influx, activates alveolar macrophages and perivascular lymphocytic aggregates over time due to the presence of endotoxin and other antigens present in the OD, such as lipopolysaccharide (LPS) and peptidoglycan (PGN) [4,67]. The aggregation of these immune cells in association with epithelial cells results in production of pro-inflammatory cytokines and release of mucin glycoproteins, which further cause inflammation by activating T cell-induced cytotoxicity, activation of B cells, or indirectly via further activation of macrophages and activation of inflammatory cascade [4,63]. The changes in the lungs’ pathology, hypersecretion of mucus compounded by impaired clearance due to poor ciliary function by ciliary cells, occlusion of distal airway, and an ineffective cough due to weak respiratory muscles clearance results in airflow restriction and progressive deterioration of lung function causing CB. Studies have demonstrated the central role of neutrophils in promoting over production of mucus and release of tissue-damaging proteases that drive CB [68,69]. However, the function of macrophages and lymphocytes and associated signaling pathways in OD-induced CB in still not fully understood. Understanding how macrophages and lymphocytes play role in developing CB will inform novel ways to delay/treat this condition.

The mechanistic hypothesis for the development of organic dust-associated CB is that during the defense against inhaled OD, the activated immune cells promote chronic inflammation, the produced oxidants damage the airway via oxidative stress, the repetitive injury or repair mechanism leads to collagen deposition and scarring, which leads to fibrotic lungs over time, and all these changes limit the airflow in the lungs developing CB [3]. Thus, this review aims to illustrate the role of macrophages and lymphocytes in CB induced by OD in agricultural workers. This review describes the responses of macrophages and lymphocytes to OD inhalation/exposure and involved signaling pathways, and how their responses lead to the development of organic dust-associated chronic bronchitis.

## 2. Methodology

### 2.1. Literature Search

For this review, an electronic literature search was done from 23 October to 2 November 2025, in several major databases, PubMed, Science Direct, Scopus, and Web of Science. The keywords used for PubMed were “(“Bronchitis, Chronic”[Mesh]) AND “Macrophages, Alveolar”[Mesh]”, “((“Agricultural Workers’ Diseases”[Majr]) OR “Lung Diseases”[Majr]) AND “Macrophages, Alveolar”[Majr]”, “((“Agricultural Workers’ Diseases”[Majr]) OR “Lung Diseases”[Majr]) AND “Lymphocytes”[Majr]”, “(“Forced Expiratory Volume”[Mesh]) AND “Bronchitis”[Mesh]”, “(“Bronchitis, Chronic”[Mesh]) AND “Leukocytes”[Mesh]”, and “(“Bronchitis”[Mesh]) AND “Dust”[Mesh]”. The keywords used for Science Direct were “bronchitis AND dust AND (macrophage OR lymphocytes)”, “chronic bronchitis AND dust AND leucocyte”, “airway obstruction AND swine dust”, and “swine dust AND lung disease”. The keywords used for Scopus were “bronchitis AND dust AND (macrophage OR lymphocytes)”, “chronic bronchitis AND dust AND leucocyte”, “airway obstruction AND swine*”, and “(organic dust OR swine dust OR agricultural dust)” AND “lung disease AND immune cells.” Keywords used for Web of Science were “bronchitis AND dust AND (macrophage OR lymphocytes)”, “bronchitis AND (organic dust OR swine dust OR agricultural dust) AND (leucocytes OR immune cells)”, “(organic dust OR swine dust OR agricultural dust) AND lung disease AND immune cells”, “(organic dust OR swine dust OR agricultural dust) AND (lung disease OR airway obstruction) AND immune cells”, “bronchitis AND (B cell or T cell or lymphocyte or macrophage) AND (organic dust OR swine dust OR agricultural dust)”, and “(bronchitis or forced expiratory volume) AND (B cell or T cell or lymphocyte or macrophage) AND (organic dust OR swine dust OR agricultural dust).”

**Figure 2:**
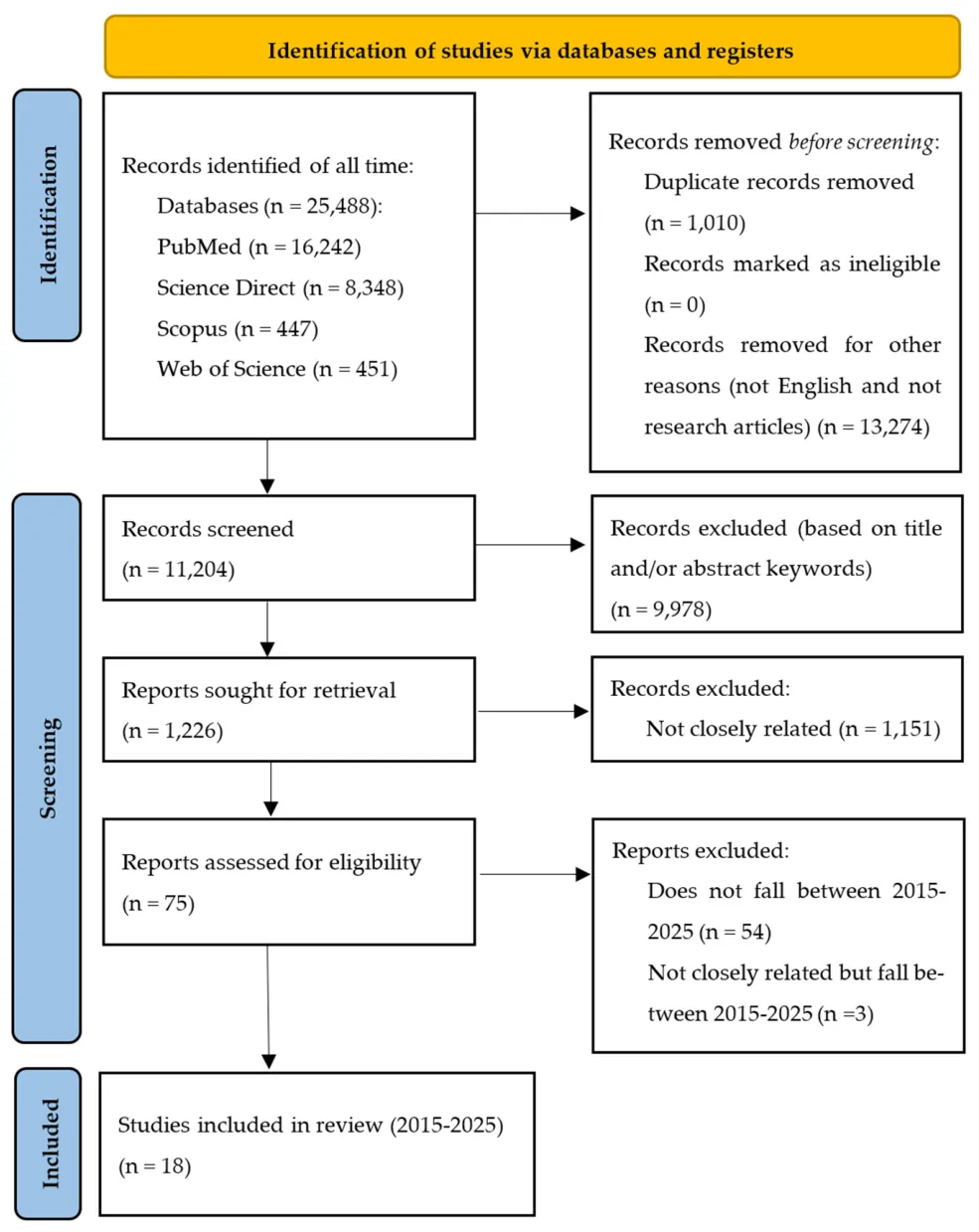
PRISMA flow diagram of the literature search and article selection process.

The initial search for all time resulted in a total of 25,488 hits combined from all four databases. After removing articles that are not English and filtering research articles only, the total hits were reduced to 12,215. Out of 12,215 hits, 1010 duplicates were removed, 9978 were removed due to irrelevance to the topic based on title/abstract, 1151 were removed because they were not closely related to the topic, and 57 articles were removed because they were before 2015 (n = 54) and not closely related (n = 3). Therefore, the most recent 18 articles (published between 2015 and 2025) were selected for this review. The Preferred Reporting Items for Systematic Reviews and Meta-Analysis (PRISMA) flow diagram shows the literature search and selection criteria applied for this review (Figure 2). The findings of the articles were determined and described based on the PICO framework as Population (human, mouse, and cell), Intervention (organic dust/organic dust extract, lipopolysaccharide), Comparator (macrophage and lymphocyte populations and duration of exposure), and Outcome (changes in macrophages, lymphocytes, and cytokines production).

### 2.2. Inclusion and Exclusion Criteria for Research Articles

Research articles published in four databases in English included in this literature analysis were studies conducted using human, mouse, and cell line models and aligned with the keywords, and timeframe (2015–2025). Case reports, books and documents, clinical trial, meta-analysis studies were included in the search. Articles published in other language than English and review articles were not included in this study. Using the process illustrated in Figure 2, the selected research articles were transferred to a spreadsheet and organized by keywords including bronchitis/chronic bronchitis, OD or LPS or PGN, macrophage, and lymphocyte in the title or abstract were included leading to 1,226. Articles that were removed were focused on diseases including asthma, pneumonia, lung carcinoma, cystic fibrosis, and lung injury. From the curated list of 1,226 papers, 75 were deemed to be closely related to the topic. To include only the most recent studies, articles published from 2015 to 2025 were included, resulting in 18 research articles. The models used in those 18 articles involved use of humans, mice, and cell lines. Agreement upon which models to focus along with the article identification and screening process was the consensus of all authors (Figure 2).

Framework for this review was prepared by two authors (J.P.R. and J.L.G. Jr.), articles were searched using criteria mentioned above, screened, discussed, and selected by two authors (J.P.R. and J.T.W.), and concepts of immunology by two authors (J.P.R. and R.C.M.) independently to avoid risk of bias. Any disagreements were resolved by two authors after discussing with second (J.L.G. Jr.) and third author (R.C.M.). The following items were extracted from the selected articles: objectives/hypothesis, sensitization or exposure sequence, methods: assays, macrophages related findings, lymphocytes related findings, cytokines, and signaling pathways. The extracted information from the articles was synthesized, tabulated based on the model used in each study, and agreed upon by all authors (J.P.R., J.L.G. Jr., R.C.M. and J.T.W.). These articles were critically analyzed for themes that may contribute to understanding of the role of lymphocytes and macrophages in development of CB in the context of OD exposure. Additional supporting literature has been included to position in the work in the field.

## 3. Results of Literature Analysis

### 3.1. Organic Dust Stimulated Macrophages and Lymphocytes Response Using Human, Mice, and Cell Lines

From the selected articles (n = 18), 11 studies used mice as a model, one (1) study was based on human + cell line, one (1) study was based on human + mice + cell line, and five (5) studies were based on mice + cell line. Among the mice only studies (n = 11), nine (9) studies used C57BL/6 mice, one study used C57BL/6J mice, and one study used C57BL/6N mice, and mice were exposed to 12.5% extract OD for one day (acute) to 3-week (chronic) exposure. Mice were also exposed to LPS alone (n = 1) or LPS with cigarette smoke (n = 1). One human study (n = 1) assessed patients with CB and another human study evaluated veterans with COPD, which was extended to in vivo and in vitro models (n = 1). To evaluate the various objectives, cell lines used in the studies were murine alveolar macrophage cell line (MH-S cells), THP-1 human monocytes, NCI-H292, primary mouse alveolar macrophages, and primary human monocytes. If the studies included OD components such as LPS, those studies and their combined treatments were also reported in Table 1 and Table 2. Common methods used in the studies were ELISA, H&E staining, RT-PCR, immunoblotting, pathology scoring, and flow cytometry. Other methods were NanoString technology, UPLC-MS/MS, and FEV1 measurement. The details about objectives, sensitization or exposure sequence, methods or assay used, major findings about macrophages and lymphocytes, expression of cytokines, and involved signaling pathways are shown in Table 1 and Table 2 with the report of mice only studies (Table 1) and studies involving on human, mice, and cell lines or together (Table 2). For the sake of this review, single or 1-day exposure is considered as acute and 2 or more days of exposure is considered as repetitive or chronic exposure in mouse model.

### 3.2. Signaling Pathways

Organic dust stimulation of macrophages and lymphocytes resulted in an increase in proinflammatory cytokines and airways responsiveness via multiple signaling pathways, which resulted into either resolution or disease condition. The involved signaling pathways are shown in Table 1 and Table 2 in the selected articles. Most common pathways observed in studies were NF-κB, PKC, TLR4, TLR2, and ERK pathways (Table 1 and Table 2). Other pathways involved were TLR/MyD88, p38, p-c-Jun, IκB, and p53 pathways. In the articles, if studies were conducted in other cell lines such as epithelial cells along with mice model exposed to OD or its components, and signaling pathways were reported, they are also mentioned in Table 2. Several studies did not mention the signaling pathways involved. In such cases, the produced cytokines and responses of macrophages and lymphocytes to OD and its components were reported and may implicate signaling pathways.

## 4. Repetitive Organic Dust Exposure, Acute and Chronic Lung Inflammation, and Implications for Organic Dust-Associated Chronic Bronchitis

### 4.1. Airway Inflammation, Cytokine Production, Cell Influx, and Tissure Remodeling in Response to Organic Dust Exposure

The prevalence of CB, accompanied by a reduction in forced expiratory volume (FEV1), is higher among livestock farmers, especially swine workers, due to repeated OD exposure in the swine confinement buildings [6,82,83]. Organic dust present in agricultural farms contain microbial components (lipopolysaccharide, peptidoglycans, fungal β-glucans, etc), fecal matter, and feed particles [84]. Acute or chronic exposure to OD results in activation of inflammatory response, recruitment of immune cells including alveolar and interstitial macrophages and T- and B-lymphocytes in the lungs, resulting in decrease in lung function via multiple signaling pathways [29,70,71,72,75].

Repetitive ODE exposure increases macrophage and lymphocyte influx into bronchi and alveolar spaces, mucus cell hyperplasia/metaplasia, and formation of lymphoid aggregates in mouse models, features which align with signs of chronic bronchitis [31,70,72]. Organic dust exposure induces inflammation through the recruitment and action of monocytes/macrophages along with neutrophils, airway epithelial cells, and innate lymphoid cells [15]. Alveolar macrophages (AMs), innate immune cells located in close contact with epithelial cells in the alveolar space, are the first sentinels in the respiratory system to recognize pathogens through receptors such as pattern recognition receptors (PPRs), and help to clear the pathogens, allergens, and inhaled dust, thereby activating the inflammatory cascade and other immune cells and thus maintaining pulmonary homeostasis [85,86,87]. Both acute and continuous or repetitive exposure to ODE increases macrophages and macrophages associated markers in the lungs of exposed individuals [31,72] A general theme of the reviewed studies is that immune cell recruitment is a marker of inflammation. Cell influx upon repetitive exposure may be associated with resolution phase or adaptation response, conceivably to clear early responder cells, dust particles or dust components in the context of chronic exposure, and yet cell influxes may not always be associated with pathological changes [70,72,74]. Though many studies measured cell influx, only limited studies described activation state, such as macrophage polarization/differentiation. Acute OD exposure did not change macrophage influx in animal models [31]. However, chronic OD exposure in animals increased macrophages in BALF and lung tissue [2,18,29,63,72,73].

In healthy humans, swine barn dust exposure for few hours increased monocytes count and pro-inflammatory cytokines and chemokines levels in sputum and blood, i.e., increase in total cells count, IL-6, TNF-α, matrix metalloproteases (MMP)-9, TIMP-9, IL-8, and LTB_4_ in sputum and monocytes count, neutrophils count, IL-6, and MMP-9 in blood after exposing to swine barn dust [88]. IL-6 could reduce macrophage death and thereby help in reducing lung inflammation [89]. However, the dual nature of IL-6, both pro- and anti-inflammatory, does not make IL-6 always helpful in reducing lung inflammation [90]. Enhanced expression of MMP-9 and TIMP-1 indicates tissue degradation, whereas, LTB_4_, a mediator for cell migration that attracts neutrophils to the site in airways, are collectively found in acute COPD cases [88]. One source of MMP-9 is macrophages, where MMP-9 is type IV collagen-degrading MMP and plays a role in lung repair via participating in inflammatory processes [88]. MMP-9 and TIMP-1 concentrations are higher in sputum of CB patients and together, they play a role in ECM remodeling and airflow obstruction; however, this was not reported in the selected articles nor correlated with influx of macrophages [91].

In CB patients, macrophages were higher in sputum with increase in neutrophils, lymphocytes, and eosinophils and blood tests showed increase in leukocytes. In WT mice, three weeks of organic dust extract (ODE) exposure elevated levels of neutrophils, macrophages, and lymphocytes count along with increase in TNF-α, IL-8, CXCL1 (or KC), and CXCL2 (or MIP-2α) in bronchoalveolar lavage fluid (BALF) [72]. CXCL1, CXCL2, CXCL3, CXCL5, CXCL6, and CXCL8 are chemokines, and CXCL2 (or MIP-2α), produced by activated macrophages, binds and activates neutrophil via CXCR2/PKC/NOX4 pathway and causes tissue damage [92]. In addition, ODE exposure also increased IL-33 in lungs and serum IgE level [72]. The pathological changes in airways and lungs caused by OD exposure were mucus cell metaplasia, epithelial hyperplasia, and persistent perivascular lymphoid infiltrates [72,77]. Lipopolysaccharide (LPS), a component of OD, exposure also increased numbers of macrophages, lymphocytes, and neutrophils in BALF with LPS alone or together with cigarette smoke extract [72,76,77,80,93]. LPS alone or together with cigarette smoke extract increased pro- and anti-inflammatory cytokines and chemokines such as TNF-α, IL-6, MIP-1α, CCL5, CXCL2, CXCL5, as well as MPO, PAS, MUC5AC, MUC5B, elastase, and ROS in mouse and cell models [75,76,80,93]. Pathological changes such as goblet cell hyperplasia, collagen deposition, oxidative stress, and mucus hypersecretion were caused by exposure to LPS or LPS with cigarette smoke extract [79,80,93]. When considering these reviewed studies together, the increase in immune cells influx, inflammatory mediators along with pathophysiological changes results in chronic inflammation, and thus, demonstrates an association between organic dust exposure and chronic inflammation in lungs.

### 4.2. Diet, Macrophages, and Organic Dust-Induced Lung Inflammation

Dietary intervention alters the inflammatory effects of OD or LPS exposure. Foundational work on this topic demonstrated that vitamin D reduced organic dust-induced airway inflammation, a hallmark of CB, with low vitamin D levels associated with high risk [94,95]. Repetitive ODE exposure in mice fed a dietary component, e.g., bovine milk-derived extracellular vesicles, showed decrease in M2 macrophages (anti-inflammatory) associated cytokine, IL-10, and increase in M1 macrophages (pro-inflammatory macrophages) associated cytokines such as TNF-α, IL-6, and IL-12/23 compared to diet containing disrupted extracellular vesicles [18,72]. Upon single exposure to ODE, mice maintained on a zinc deficient diet showed increased total cells count and neutrophil count compared to control (saline-exposed zinc deficient diet fed mice), and similarly, ODE also increases IL-6, TNF-α, CXCL1, and AREG compared to zinc sufficient diet fed ODE-exposed mice [81]. Whereas, repetitive ODE exposure (for three weeks) with zinc deficiency increases macrophages and total cell influx in bronchoalveolar lavage fluid (BALF), along with increased levels of inflammatory cytokines and chemokines such as IL-6, TNF-α, CXCL1, and AREG, P65 protein activity, and decreased activation of protein kinase C (PKC) isoforms [81]. ODE exposure also affected blood macrophage function, for instance, ODE exposure increased both pro- and anti-inflammatory cytokines such as TNF-α, IL-1β, IL-6, and IL-8 when exposed to blood macrophages with zinc deficiency [96]. Such studies show the importance of micronutrients in the diet, which helps to lower the immune cells influx and inflammation in lungs. However, a diet containing docosahexaenoic acid (DHA), a polyunsaturated omega-3 fatty acid that has anti-inflammatory properties, containing diet followed by one week ODE exposure showed no change in macrophages level in BALF of mice [73]. Macrophages were still higher in BALF after allowing 7 days recovery [73]. This observation suggests that prolong ODE exposure has lasting effects on the lungs, even after stopping the exposure. The specialized pro-resolving lipid mediator maresin-1 (MaR1) was shown to be effective in acute and repetitive ODE exposure reducing neutrophil infiltration, IL-6, TNF-α, CXCL1, and lung tissue ICAM-1; however, macrophages influx remained unaffected [30]. An increase in CXCL8 is associated with inflammation in human via JAK-STAT pathway [97]. Chemokine CXCR1/CXCR2 antagonist, CXCL8/K11R/G1P (G31P) has also been shown to be effective at reducing neutrophil influx, IL-6, KC, MIP-2, ICAM-1, CXCR1, and CXCR2, PKCα, and PKCε were reduced with G31P treatment with ODE exposure [29]. Therefore, pro-resolving lipid mediator (MaR1) and chemokine antagonist (G31P) are shown to be effective compared to allergen (ovalbumin) sensitization and IL-22 knockout in mediating ODE-induced inflammatory signaling pathways and macrophages influx. Based on this collection of reports, there is an association between the immune cellular and molecular response to OD exposure (such as CXCR1/CXCR2 binding with CXCL8) and changes in the lung pathology, indicating that byproducts of OD exposure may be worse than OD exposure itself [29]. Consumption of a macronutrient- and micronutrient-rich diet, such as containing DHA and zinc, may help to protect the lungs against the effects of repetitive OD exposure [73].

### 4.3. Antioxidants and Anti-Inflammatory Compounds

Antioxidant and anti-inflammatory agents help to mitigate OD-induced activation of macrophages, inflammation, and downstream signaling pathways. Use of the bioactive compound polyacetylene, a component of leaves from *Dendropanax morbiferus* (*D. morbifera*)—commonly known as the Hwangchil tree or Korean *dendropanax*, has anti-inflammatory and antioxidant properties, and was effective at reducing combined effect of LPS and cigarette smoke extract such as reduction in macrophages influx, IL-6, TNF-α, ROS, and elastase in mice [79]. Another antioxidant and anti-inflammatory agent, phycocyanin (bioactive compound found in blue-green algae like *Spirulina*), was able to reduce lung inflammation by reducing the inflammatory cell influx, goblet cell metaplasia, and collagen deposition in cigarette smoke extract and LPS-exposed mice [93]. Phycocyanin regulated heme oxygenase 1 (HO-1) and NADPH dehydrogenase quinone (NQO1) level in lung and NOX2 in pulmonary macrophages exposed to cigarette smoke extract and LPS [93]. In addition, phospho-NF-E2-related factor 2 (Nrf2) and NQO1 protein expression are induced by cigarette smoke extract in RAW264.7 was reduced by phycocyabilin–phycocyanin–peptide [93]. Other compounds, such as eucalyptol, limonene and pinene enteric capsules (ELPs), which are mucolytic agents extracted from plants of Myrtaceae, Rutaceae, and Pinaceae families, reduced levels of macrophages, neutrophils, and total cells influx with reduction of IL-6, TNF-α, MIP-1α, and CCL5, similar to the effect of dexamethasone administration [80]. ELP and dexamethasone also reduced PAS, MUC5AC, MUC5B in LPS-exposed animal models [80]. Moreover, a synthetic copy of monoacetyldiaglyceride (1-palmitoyl-1linoleoyl-3-acetylglycerol, PLAG—a constituent found in sika deer and known for immunomodulation), EC18, reduced cigarette smoke extract and LPS-induced ROS, elastase, IL-6, TNF-α, and MPO in the BALF of animals with a reduction in pp65 and p-IκB. Therefore, antioxidant and anti-inflammatory agents are effective at reducing immune cell influx (especially neutrophils and macrophages), pro- and anti-inflammatory cytokines, mucus secretion, ROS, and inflammation, minimizing the pathophysiological changes due to OD, LPS, and cigarette smoke extract exposure in both cell line and animal model.

### 4.4. Lymphocyte Influx in Response to Organic Dust Exposure

Lymphocytes influx was increased in response to repetitive ODE exposure with increase in lymphoid aggregates in mice and their influx varies in acute exposure [18,72,73,81]. In chronic OD exposure conditions, once the lymphocytes influx increases, levels of these cells remain high even after allowing a week-long recovery period in ODE-exposed mice [73]. The response was independent of sex in ODE challenged mice [77]. Mice sensitized with ovalbumin showed higher lymphocytes count, whereas, OVA + ODE exposure increased CD3^+^ T cells, CD19^+^ B cells, and NK cells compared to ODE exposure alone, which indicated the potentiated inflammatory response upon co-exposure of ODE with ovalbumin allergen [74]. Similar to ODE, LPS treatment of C57BL/6J mice followed by cigarette smoke exposure increased lymphocyte numbers compared to control [93]. However, LPS alone showed variable results, for instance, in Wister rats, LPS exposure for increasing amount of days (0, 7, 14, 21, 28, 35, 42, and 49 days) showed no difference in lymphocytes count; however, one-time LPS exposure in C57BL/6J mice showed increase in lymphocytes count (increase in CD3^+^ T cells, CD4^+^ T cells, and CD8^+^ T cells) compared to control [76,80]. The difference in outcome could be due to repetitive vs. single (short-term) exposure, which could lead to reduction in sensitivity over time or perhaps difference in genetic background between the models. The results of studies that use inbred strains of mice or rats should always be carefully evaluated because the differences in genetic background can have profound impacts both within and between species. B cells receptor knockout (BCR KO) mice exposed to ODE showed reduction in the inflammatory cytokines and chemokines such as TNF-α, IL-6, CXCL1, CXCL2 release with compensatory increase in T cells [63]. In humans, in vivo exposure to swine barn dust for two hours showed reduction in lymphocytes in healthy subjects and no change in lymphocytes counts [88]. These results support the fact that long-term exposure to OD and its components promote decreased sensitivity of lymphocytes and lead to prolong unresolved inflammation in the lungs. Additional mechanistic studies that define signaling pathways involved in regulating lymphocyte action in organic dust-associated chronic bronchitis will help to resolve discrepancies.

Acute LPS exposure (0.5 mg/mL for 24 h) increases chemokines that activate lymphocytes, including CXCL2 (associated with neutrophil chemoattractant and Th17), CXCL5 (RANTES; associated with B lymphocytes and Th17), CCL2 (associated with Th2), CXCL10 (associated with Th1 lymphocytes), and cytokines such as TNF-α and IL-6 with an increase in NK cells [76,80]. TNF-α is produced by both lymphocytes and macrophages, and the function of TNF-α is both pro- and anti-inflammatory, which demonstrates a duality of roles by cytokines [98]. Furthermore, with repetitive LPS exposure, there was no difference in lymphocytes count, but there was increased levels of CCL5, TNF-α, IL-6, and MIP-1α (chemoattractant for macrophages and other inflammatory cells; characteristics of CB) in the BALF of Wistar rats [80]. Together, OVA sensitization and 4–5 h exposure to 12.5% ODE caused C57BL/6J mice to show increased levels of airway T cells, B cells, and their markers/regulators such as CXCL1 (associated with Th17), CXCL2, CCL2, CCL3 (MIP-1α), CCL12, CCL22 and cytokines such as IL-6, IL-4, IL-13 compared to control (saline) mice but no detection of IL-9, IL-17A (produced by Th17 cells and NK cells), IL-17E, IL-33, and CCL8 [74]. IL-4 and IL-13 are produced by Th2 cells, and their increased level is associated with persistence of airway inflammation [99]. T and B lymphocytes generate antigen specific memory cells (CD4) that are quickly reactivated upon re-exposure. However, continuous presence of antigens causes prolonged activation of lymphocytes and generation of cytokine storm, and when there is failure to resolve the inflammatory damage then the lung function starts to fail causing respiratory diseases [63].

### 4.5. Diet, Lymphocytes and Organic Dust-Induced Lung Inflammation

**Figure 3:**
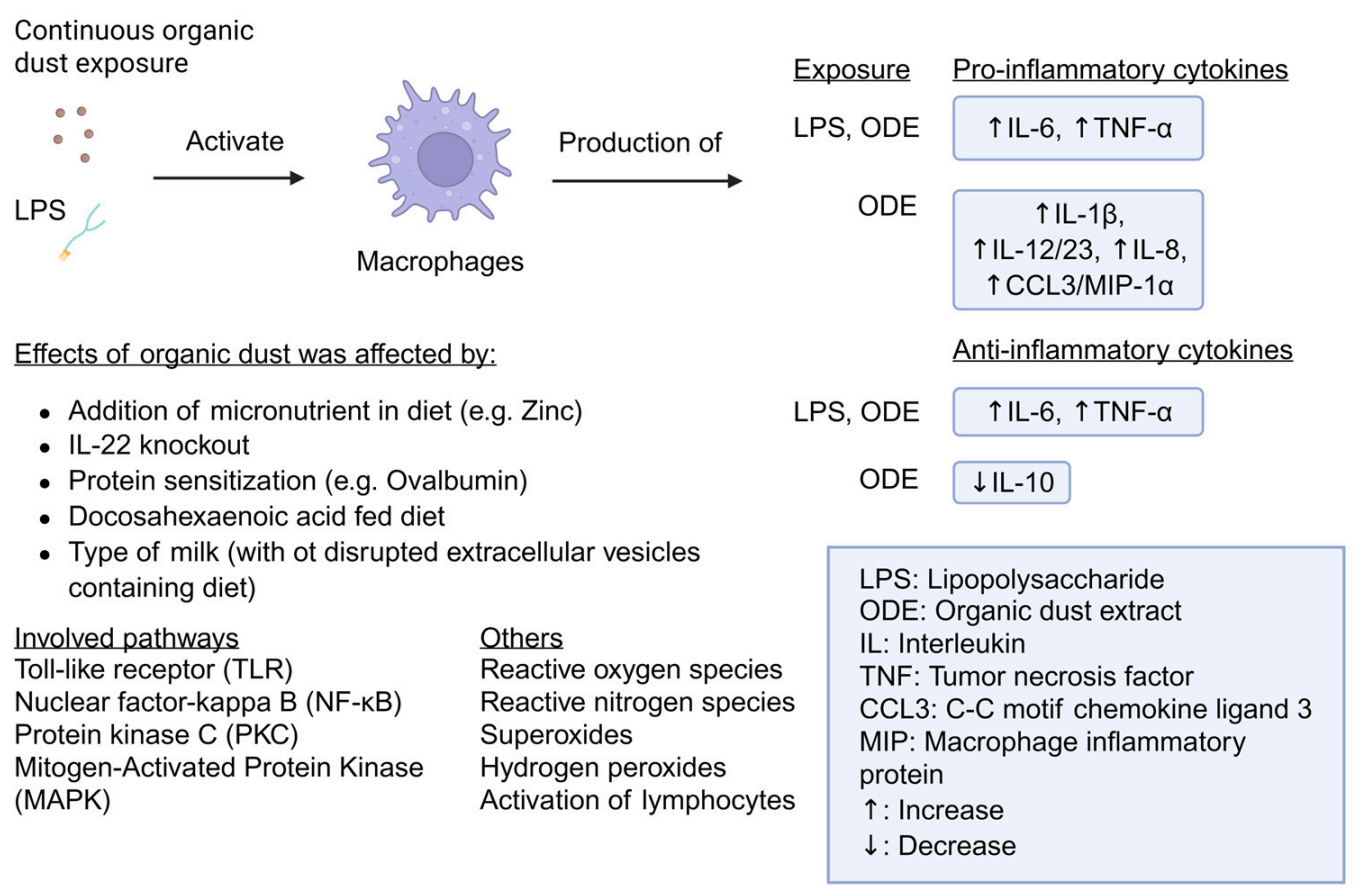
Summary of the literature showing effect of organic dust on chemokines and cytokines and activation of lymphocytes. (Created with https://BioRender.com).

Studies have been done to investigate if altering the diet or using the knockout of major molecule/protein could mitigate the lymphocyte mediated inflammation. The literature summarizing the effect of OD and LPS, on the production of chemokines and cytokines, and regulation of lymphocytes is shown in Figure 3. Changing the diet and knocking out certain proteins showed little to moderate impact on lymphocytes level when mice are exposed to ODE. For instance, repetitive ODE exposure increased lymphoid aggregates in mice fed an extracellular vesicle-enriched (EV) diet compared to mice fed a disrupting extracellular-vesicle (DEV) diet [18]. However, detailed studies on lymphocytes and their subsets were not done in this study. In C57BL/6 mice that were fed a docosahexanoic acid (DHA) enriched diet, there was increase in lymphocytes after 12.5% DE exposure for three weeks similar to control; however, no difference in lymphocytes levels due to the DHA enriched diet was observed [73]. TLR adaptor protein myeloid differentiation factor 88 (MyD88) KO mice showed that saline pretreatment followed by ODE exposure increased lymphocyte numbers compared to ODE pretreatment followed by exposure to ODE, thus showing the importance of MyD88 in regulating the lymphocyte action in OD-induced inflammation in the lung [73]. With the exception of a few studies explaining responses to OD exposure, there is limited evidence defining the role of lymphocyte populations of OD-exposed individuals and development of CB [18,73].

### 4.6. Signaling Pathways Associated with Organic Dust-Induced Lung Inflammation

Organic dust or its components (e.g., LPS), after entering into the lungs, is recognized by TLR4 that is present on the immune cells, which activate the NF-κB signaling pathway [100]. LPS exposure showed increase in phosphorylation of ERK 42kDa, IRF3, p65, and c-Jun in rats [80]. In vitro studies with BEAS-2B cells (human bronchial epithelial cells) show similar results, with increased TLR4 signaling in LPS-exposed cells. Similar observations were reported for C57BL/6N mice exposed to cigarette smoke followed by LPS treatment [75]. Mice with or without p53 knockout showed that LPS exposure in p53 mice was able to reduce influx of immune cells as well as pro- and anti-inflammatory molecules and cytokines which also indicated that involvement of p53 pathway in LPS-exposed cases [76]. Another study demonstrated that LPS exposure activated MAPK signaling via ERK and p38 signaling in mice [79]. Pro- and anti-inflammatory markers such as TNF-α, IL-1, IL-6, and macrophage inhibitory factor (MIF) are activated and regulated by the NF-κB pathway confirming that OD exposure increases inflammation via the NF-κB pathway [31,81]. TNF-α binds to TNF-α receptors 1 and 2 (TNFR1 and TNFR2), and these receptors are known to bind with c-Jun N-terminal kinases (JNKs) and p38 MAPK, which in turn activate transcription of many genes via transcription factors (e.g., AP-1) [101]. Furthermore, TNFRs activate the ERK signaling pathway through activation of the TPL2-MEK-ERK (Tumor Progression Locus 2 (TPL2)-Mitogen-Activated Protein Kinase (MEK)-Extracellular Signal-Regulated Kinase (ERK)) pathway, leading to activation of the NF-κB pathway and downstream apoptosis [101]. The protein kinase C (PKC) pathway also plays a role in mediating OD-exposed inflammation. Exposure of tracheal epithelial cells and lung tissues to swine barn OD showed increase in PKCα, PKCε, and PKC*ξ* in C57BL/6 mice showing that PKC pathway involvement [29,81]. Proteases present in OD were reported to be important drivers of ODE-induced inflammatory processes via protease-activated receptors (PARs; G-protein coupled receptors) signaling and depletion of protease (and PAR-1 and 2 genetic deficiency) reduced the activation PKCα and PKCε in bronchial epithelial cells and mouse lungs [102]. Crosstalk between PKC and NF-κB signaling is demonstrated by PKC-mediated increase in proinflammatory cytokines such as IL-1β expression via NF-κB pathway in LPS-induced inflammation [103,104]. The addition of anti-inflammatory drug (dexamethasone), salbutamol, Zn sufficient diet, lipid mediator maresin-1 (MaR1) pretreatment, bovine milk-derived extracellular vesicles containing diet, use of antioxidants (e.g., sorrel dust extract), or alteration of receptor such as use of CXCR1/CXCR2 antagonist, help to reduce the DE-induced inflammation [18,29,31,81,105]. Thus, there are multiple intersecting pathways associated with OD-induced inflammation that may help to explain CB development in agricultural settings.

### 4.7. Perspectives on the Current Literature Describing Organic Dust-Associated Chronic Bronchitis

Most of the studies in the collection of literature analyzed herein focused on NF-κB and PKC pathways in the context of OD exposure and airway inflammation. Therefore, further exploration into the roles of MAPK, JAK-STAT, and JNK signaling pathways is needed to understand the mechanistic hypotheses orchestrating development of organic dust-associated chronic bronchitis in agricultural workers.

It is well documented that macrophage-lymphocyte crosstalk mediate lung inflammation, fibrosis and other respiratory health effects through positive feedback that generally involves communication largely through cytokines/chemokines. In the context of reports informing this review, macrophage-lymphocyte crosstalk is achieved via cytokine signaling loops where macrophages present antigens and secrete cytokines/chemokines that recruit and polarize T cells via TLR signaling. T cells in turn release cytokines (including interferons) that promotes activation and differentiation of macrophages toward the inflammatory (M1) or tissue remodeling and fibrotic (M2) phenotypes in response to inflammatory compounds, such with organic dust exposure as reported in studies mouse models and reviews of human and rodent studies [106,107,108].

Methods used to collect data for human studies included interviews, questionnaires, measurement of forced expiratory volume (FEV1), RNA analysis, and proteome profiler assays [33]. Other methods involved sputum and BALF analysis, pulmonary function tests. In recent years, there have been limited reports on studies of swine farmers exposed to organic dust, which not only limits the availability of knowledge about mechanisms of organic dust-associated chronic bronchitis and whether there have been recent interventions such changes in facilities or management practices and outcomes on lung health. Cell culture and animal studies have helped to fill some of the gaps mentioned here. Mouse models provide insights for understanding OD exposure in humans through histopathological scoring, immunostaining, flow cytometric analysis, and specific gene knockout [70,71,72,74,76]. For instance, insufficient zinc intake is associated with a reduction in pulmonary function in humans and in zinc deficient mice, there was increased hyper-responsiveness mediators (IL-6 and TNF-α) and pronounced airway inflammation [81]. However, care must be taken when translating findings from preclinical mouse studies as they may not adequately predict human clinical outcomes because of fundamental biological differences and variability of study designs. Despite the limitations of in vitro cell and preclinical mouse models, they represent an extensive and powerful collection of tools for genetically controlled, mechanistic studies, screening and related early stage studies. Thus, the animal models or cell studies reviewed herein only provide potential mechanisms for understanding organic dust-associated chronic bronchitis in humans. Importantly, human cell culture studies remain essential for modeling the effects of OD on the lungs. The application of integrated NAMs for investigating the complexities of human environmental and occupational exposures, surveillance, interventions (both workplace and therapeutic), and in vitro studies represent a potential path toward delving into deeper mechanistic understanding and predicting outcomes for organic dust-associated chronic bronchitis.

This literature analysis has several limitations that must be defined for context. The review focused on literature reports related to OD and its component’s (LPS) exposure within a limited time frame 2015–2025 providing the summary of findings on the effect of OD in lungs and associated pathways. However, this review does not extensively include reports of studies published beyond that time frame or explore every possible component of OD. This study is focused on the effect of OD, especially swine barn OD; therefore, it does not include the effect of inorganic agricultural dust on respiratory health of farm workers [109,110]. Primarily, two types of immune cells—macrophages and lymphocytes—and their association with inflammation and inflammatory markers upon OD exposure were mainly discussed in this study. Moreover, exclusion of other databases may have missed information unique to those databases. However, this review provides valuable insights for understanding responses of macrophages and lymphocytes upon OD exposure and resultant lung inflammation. This review has shown the relationship between expression of chemokines and cytokines with lymphocyte subtypes. Further wet bench studies could be performed to further define the role of T and B cells in organic dust-associated chronic bronchitis. Moreover, the studies reviewed herein focused on responses of immune cells of lungs (mainly macrophages) to organic dust or LPS exposure alone or LPS exposure along with other treatments such as cigarette smoke extract and explained the relationship between alveolar macrophages and lymphocyte based on the inflammatory markers. However, limited studies have reported macrophage–lymphocytes crosstalk, which presents an opportunity for new approach methodologies (NAMs) such as three-dimensional co-cultures, microfluidic devices, organoids, organ-on-a-chip and computational model to define macrophage-lymphocyte crosstalk to investigate dynamic cell signaling. The studies were mostly focused on cell lines and small animals, such as mice and rats. Future studies could be expanded to include lemur studies and larger animal models, such as pig macrophages and lymphocytes, or phylogenetically closer animals, such as primates.

## 5. Conclusions

Repetitive exposure to organic dust is associated with the development of chronic bronchitis in agricultural workers. The OD exposure results in increase in total immune cells, macrophages and lymphocytes along with neutrophils and eosinophils depending upon short or long-term exposure. These reviewed studies suggest that chronic OD exposure induces the activation of macrophages and lymphocytes for a longer period, which results in activation of multiple signaling pathways resulting in chronic airway inflammation. The constant activation of macrophages and lymphocytes causes excessive generation of pro- and anti-inflammatory markers in the lungs and the crosstalk between macrophages and lymphocytes increases those markers. This leads to excessive generation of inflammatory cytokines and chemokines, increase in cell influx, oxidative stress, impairment or adaptation of macrophages and lymphocytes, and inability to resolve the inflammation over time. Eventually, the airway compensates by replacement of ciliary epithelial cells with squamous cells, and collagen deposition in the lungs leading to fibrosis developing CB. This study also highlighted how the alteration in diet or pathways could change the expression of macrophages and lymphocytes and modulate lung inflammation via PKC, NF-κB, and MAPK pathways. Thus, this study provides mechanistic hypotheses involved in organic dust-associated chronic bronchitis in agricultural workers.

## Figures and Tables

**Table 1:** Functional response of alveolar macrophages and lymphocytes due to organic dust extract exposure and lipopolysaccharide in mice and involved Signaling pathways.

References	Objectives/Hypothesis	Sensitization, Route, Dose, or Exposure Sequence	Methods: Assays	Macrophages Related Findings	Lymphocytes Related Findings	Cytokines	Signaling Pathway
**Dean et al., 2025** [70]	Impact of omega-3 fatty acids on the comprehensive immune cell populations following OD exposure	C57BL/6 WT mice and Fat-1 transgenic C57BL/6 mice >> ODE or PBS control for 3 weeks >> euthanize after 5 h post treatment. **Exposure:** Intranasal, 12.5% ODE No recovery period.	H&E staining, flow cytometry, ELISA, NanoString technology, pathology scoring	ODE ↑ monocyte and innate lymphoid cells (ILCs) populations and ↓ neutrophil populations in Fat-1 compared to WT mice. ODE did not change AMs counts in lung tissue in PBS control or ODE WT or Fat-1 mice, regardless of sex, but ↑ in interstitial macrophages in both WT and Fat-1 mice (female > male). Classical and intermediate monocyte counts ↑ in ODE exposed Fat-1 mice (female > male) than WT.	ODE ↑ B cells in WT male mice and NK cell count in WT females. CD4^+^ and CD8^+^ T cells ↑ in Fat-1 mice (female). ILC3 count: ODE exposure ↑ ILC3 counts: Fat-1 female mice > WT females < WT male mice. NCR3-ILC3 higher in male ODE exposed WT mice. ODE increases alveolar inflammation, immune cell infiltration into alveolar space, bronchioles (irrespective of sex or genotype), formation of aggregates, and metaplasia of goblet cells within bronchi.	ODE ↑ long chain-fatty acids (*Elov6*, and *Alox15*) in male mice. ODE ↑ *Ccr3* and *Ccl17* in Fat1 male mice than WT male. ODE exposed Fat-1 female mice showed ↓ in scavenger receptors (*Marco* and *Mpeg1*), ↑ in *Ccl20* and *Fgf2*, and also ↑ in MCP-1 (than Fat-1 males and WT females). CX_3_CL1 ↑ in Fat-1 male than WT male mice. ODE altered *Cxcr3*, *Retnla*, and *Ccr3* in Fat-1 mice.	N/A
**Pandher et al., 2021** [71]	Additive or synergistic effects of combination exposure (LPS and glyphosate) on lung inflammation compared to individual LPS or glyphosate exposures after both short (5 day) and longer-term (10-day) exposures	C57/BL6 mice >> 1 d, or daily for 5 d, or daily for 10 d exposure to treatments >> after 4 h of post exposure, respiratory measures undertaken >> harvest (4 h post-treatment). n = 5 mice/groups, four treatment groups: Saline control, LPS, glyphosate alone, or LPS + glyphosate. **Exposure:** Intranasal, 0.5 µg/40 µL LPS, 1 µg/40 µL glyphosate No recovery period.	PenH measures using WBP (whole-body plethysmography), Pierce BCA assay, ELISA, H&E staining, myeloperoxidase assay, immunohistochemistry, RT-PCR	PenH ↑ in 5 d and ↓ in 10 d in both LPS and LPS + glyphosate group compared to control.	After 5 and 10 d of exposure, B and T lymphocytes ↑ around perivascular regions in the lung sections of mice exposed to LPS + glyphosate. Vascular permeability increased by LPS exposure after 5- and 10 d exposures compared to 1-day exposure.	After a day of exposure (acute) to (LPS + glyphosate) ↑ IL-6 and MIP-2 than other exposures, and ↑ IL-1β in LPS + glyphosate and LPS only exposure. LPS + glyphosate ↑ IL-6, TNF-⍺, and KC than other exposures after 5 days exposures suggesting additive effects. LPS + glyphosate ↓ IL-6, TNF-⍺, KC, IL-1β, and MIP-2 compared to respective group with 5 d exposure. IL-4 ↑ in LPS + glyphosate group vs. respective group with 5 day exposure vs. LPS alone and glyphosate alone exposure. MPO ↑ after 1-day exposure and ↑ more after 5 d exposure in LPS + glyphosate group. LPS + glyphosate group showed ↑ in ICAM-1, TLR4, and TLR2 than other groups.	
**Johnson et al., 2020** [72]	Lung cellular and microenvironment response to repeated ODE exposure for 3 weeks with or without recovery period (i.e., 4 weeks)	No recovery period: Mice C57BL/6 WT or MyD88 knock-out (KO) mice >> expose intranasally (i.n.) to ODE or saline (PBS) 3 h * 3 weeks Rechallenged after recovery period: Mice WT or MyD88 KO >> expose i.n. to ODE or saline (PBS) 3 h * 3 weeks >> rest 4 weeks w/o treatments >> rechallenged once with saline or ODE **Exposure:** Intranasal, 12.5% ODE	ELISA, immunostaining, and RT-PCR	ODE ↑ macrophages in WT mice with recovery period but ↓ in MyD88 KO mice. ODE ↑ macrophages mediators (CCL2, CCL4, CCL7) in both WT and MyD88 KO mice. ODE ↑ in macrophages mediators (CCL8 (trend), and CXCL10) in WT mice.	ODE ↑ BALF lymphocytes in both WT and MyD88 KO mice. ODE ↑ lymphocytes in WT mice, but no change in MyD88 KO mice. ODE ↑ B cells in MyD88 KO mice with recovery period compared to mice with no recovery period. ODE ↑ lymphocytes mediators (CCL2, CCL4, CCL5) in both WT and MyD88 KO mice. ODE ↑ in CCL8 (trend), CCL22, and CXCL10 in WT mice.	Rechallenged with ODE ↑ TNF-⍺, IL-6, and murine neutrophil chemoattractant (CXCL1 and CXCL2) in WT control (saline) mice but ↓ in MyD88 KO mice. ODE ↑ CCL2, CCL3, CCL4, CCL5, and CCL7 in WT mice but ↓ in MyD88 KO mice in the lungs. ODE ↑ RNA expression of tight junction proteins; claudin-3 and occludin in both WT and KO (KO: with or w/o ODE) mice, ↑ claudin-4 in WT and claudin-7 in KO mice. However, protein expression was ↓ in ODE exposed KO mice compared to WT (claudin-4 and occludin). ODE ↑ IL-33 and IL-10 in the lung compared to control.	TLR/MyD88
**Nordgren et al., 2013** [30]	Effectiveness of MaR1 at limiting lung inflammation associated with acute and repetitive ODE exposure	Mice C57BL/6: Acute: Ethanol vehicle, 0.1, or 1 ng lipid mediator maresin-1 (MaR1) intraperitoneal (i.p.) injection at 18 h before and 30 min prior to a single instillation of saline or ODE >> sacrifice after 5 h Repetitive exposure: ethanol vehicle or 10 ng MaR1 i.p. 30 min. prior to every i.n. instillation of saline or ODE * 15 consecutive weekdays >> euthanize after 5 h of final ODE exposure **Exposure:** Intranasal, 12.5% ODE No recovery period.	ELISA and immunohistochemistry	ODE exposure (acute): No change in macrophages influx. Repetitive ODE exposure (15 consecutive days): ODE ↑ macrophages in 10 ng 7(S)- or 7(R)-MaR1 + DE compared to control (saline).	Repetitive ODE ↑ BALF lymphocytes in 10 ng 7(R)-MaR1 treated mice. ODE ↑ lymphoid aggregates all three groups (vehicle, 10 ng 7 (S) and (R)—MaR1 group).	ODE ↓ TNF-⍺, IL-6, and CXCL1 in the BALF of 7(S)- or 7(R)-MaR1 pretreated mice with ↓ ICAM-1.	N/A
**Schneberger et al., 2015** [29]	Effectiveness of G31P to inhibit chemokine responses in ODE exposure	Mice C57BL/6 >> treatments exposure (control, control + G31P, ODE, and ODE + G31P) once every day for 5 d a week * 3 weeks **Exposure:** Nasal, 12.5% ODE No recovery period.	Histology and immunohistochemical staining (H&E staining), PKC activity assay, and ELISA	Macrophages were higher in lung lavage fluid in all four treatments.	N/A	Little to undetectable IL-6, KC and MIP-2 in control and G31P in BALF. ODE ↑ IL-6, KC and MIP-2 but G31P ↓ each chemokine and cytokine compared to ODE alone. In bronchial epithelium of lungs, ODE ↑ CD54/ICAM-1 compared to control (saline). G31P ↓ ICAM, CXCR1, and CXCR2 in ODE exposure.	PKC pathway (G31P may block PKCα and PKCε)
**Ulu et al., 2021** [73]	Effect of dietary supplementation of docosahexaenoic acid (DHA) in reducing airway inflammation in a chronic ODE exposure setting	Mice C57BL/6 >> high DHA diet or a control diet lacking DHA given to mice * 4 weeks Acute exposure >> exposed once with ODE (5 h) Repetitive exposure >> exposed to ODE, 5 h * 3 weeks (15 ODE instillations) For recovery model, after last ODE exposure >> allowed mice for recovery * 7 d **Exposure:** Intranasal, 12.5% ODE	H& E staining, gas chromato-graphy (GC), UPLC-MS/MS, immunofluorescence staining, and ELISA	Repetitive ODE exposure (3 weeks) ↑ BALF macrophages in both control and DHA diet fed mice. ODE exposure ↑ BALF macrophage counts even after 7 d recovery period in both control and DHA fed diets. ODE ↑ macrophage M2 markers (Ym1 and Arg-1) and recovery period did not reduce those markers.	Repetitive ODE exposure ↑ BALF lymphocytes in both control and DHA diet. Recovery period ↓ BALF lymphocyte counts in ODE exposed both diets fed mice compared to without recovery period. ODE exposure ↑ lymphoid aggregate formation upon repetitive exposure with or without recovery period.	Repetitive exposure ↑ IL-6, TNF-⍺, CXCL1, AREG, and MPO (myeloperoxidase), and AREG. Recovery period ↓ TNF-⍺, IL-6, CXCL1, AREG, MIP, and MPO, and ↑ MIF (macrophages migration inhibitory factor) in ODE + DHA diet fed mice compared to saline control diet fed mice in BALF.	N/A
**Warren et al., 2019** [74]	Effect of ODE in worsening allergic airway inflammation including airway hyperresponsiveness (AHR), airway cellular influx, and release of type 2 mediators established after OVA sensitization and challenge	Mice C57BL/6 >> sensitize with 100 μL of chicken egg ovalbumin (OVA, i.p.) or saline on d 1 and 5 >> challenge with aerosolized 1% OVA on d 20, 21,22,25, 26,27, and 28 for 20 min. each day or saline control >> treated with ODE or saline 4–5 h >> mice sacrificed on d 29 (Four treatment groups: Saline-saline, saline + ODE, OVA-saline, and OVA + ODE) **Exposure:** Intranasal, 12.5% ODE No recovery period.	Cells stained with DiffQuick, ELISA, serum IgE level, histopathology (H&E staining), and flow cytometry	Ovalbumin ↑ macrophage counts in BALF of control mice (saline). In the tissue infiltrates, ODE ↑ macrophage counts in OVA treated mice compared to only ODE or OVA, in lung tissue.	OVA ↑ lymphocytes in saline control compared to saline alone. ODE ↑ lymphocytes in OVA treated mice compared to saline control. ODE ↑ CD3^+^ T cells, CD19^+^ B cells, and NK cells in OVA treated mice compared to ODE alone.	Acute ODE exposure (once) ↑ TNF-⍺, IL-6, CXCL1 and CXCL2. ODE ↑ CCL2 and CCL3. OVA alone or ODE alone ↑ IL-6, IL-4, IL-13, CCL12, and CCL22. OVA alone also ↑ IL-5, CCL3, CCL11. ODE with OVA ↑ TNF-⍺, IL-6, IL-4, IL-13, CCL2, CCL3, CCL12, CCL22, CXCL1 and CXCL2 compared to saline control, but ↓ TNF-⍺. Not detected: IL-9, IL-17A, IL-17E, IL-33, and CCL8.	N/A
**Poole et al., 2017** [63]	Importance of B cells in mediating OD-induced lung disease and immunoglobulin responses	WT C57BL/6 mice and B cell receptor knock out (BCR KO) mice >> ODE or PBS for 3 weeks >> euthanize after 5 h of final ODE exposure **Exposure:** Intranasal, 12.5% ODE No recovery period.	H&E staining, Flow cytometry, ELISA, immunohistochemistry	ODE ↑ exudative (activated) macrophages in BALF and lung tissue of both WT and BCR KO mice, but ↓ in lung tissue of BCR KO than WT.	ODE ↑ lymphoid aggregates (composed of T cell, B cell, and macrophages) in WT mice, ↑ in CD3^+^CD4^+^ and CD3^+^CD8^+^ T cells in both WT and BCR KO mice compared to PBS control. ODE ↑ B2, B1, B1a, and memory B cells in both BALF and lung tissue in WT mice. BCR KO did not affect T cells but ↓ lymphoid aggregates.	ODE ↑ IgG and IgE in serum, anti-malondialdehyde-acetaldehyde (MAA) and anti-citrullinated protein antibody (ACPA) in WT mice compared to PBS control. ODE ↑ TNF-⍺, IL-6, KC/CXCL1, MIP/CXCL2, IL-17A, and hyaluronan in both WT and BCR KO mice, whereas BCR KO ↓ TNF-⍺, IL-6, CXCL1, and CXCL2 compared to WT mice.	N/A
**Poole et al., 2015** [2]	Lung response following 1-week post-exposure recovery phase and potential regulatory role of scavenger receptor class A/CD204 (SRA)	CD57/BL6 WT mice and SRA KO mice >> ODE or PBS for 3 weeks >> euthanize or allowed to recover for a week without treatment (Rx) **Exposure:** Intranasal, 12.5% ODE No recovery period.	H&E staining, flow cytometry, ELISA	ODE (3-week) ↑ inflammatory cells and size, alveolar inflammation, and lung exudative CD11c^hi^/CD11b^hi^ macrophages (not decreased by a week recovery). 1-week recovery ↓ CD11c^hi^/CD11b^lo^ AMs in WT compared to SRA KO mice. ODE ↑ CD80, CD86, and I-A^b^ cell surface expression on exudative CD11c^hi^/CD11b^hi^ macrophages in WT mice.	ODE (3-week) ↑ lymphoid aggregates in SRA KO mice compared to WT mice and a week recovery period ↓ lymphoid aggregates in WT. SRA KO ↑ lymphocytes than WT. Repetitive ODE ↑ CD3^+^CD4^+^ T cells in SRA KO mice than WT, which ↑ after a week of recovery. ODE ↑ both T-helper(H)-17 and TH1 cell in both WT and SRA KO mice and TH-17 was higher in SRA KO even after a week of recovery.	ODE exposure followed by 1-week recovery still ↑ IL-17A in SRA KO mice than WT.	N/A
**Shin et al., 2016** [75]	Investigate the effects of EC-18 on cigarette smoke (CS) and LPS-induced lung inflammation by measuring neutrophilic infiltration, proinflammatory cytokines and protein expression to evaluate the beneficial effects of EC-18 against the inflammatory responses of COPD development	Mice C57BL/6N >> treatment; roflumilast and EC-18 exposed before 1 h prior to CS exposure >> CS for 1 h. on 3 consecutive days >> LPS exposure 1 h after final CS exposure. **Treatments:** Control, CS + LPS, ROF (CS + roflumilast), EC-30 (CS + LPS + EC-18-30), and EC (CS + LPS + EC-18-60). Positive control is roflumilast) **Exposure:** Intranasal, LPS and Cigarette smoke (CS) No recovery period.	Inflammatory cell count, intracellular ROS activity, neutrophil elastase activity, ELISA, H&E staining, immunoblotting.	N/A	N/A	CS + LPS ↑ IL-6 and TNF-⍺ in BALF compared to control, whereas ROF, EC-18-30, and EC-18-60↓ IL-6 and TNF-⍺ compared to CS + LPS exposed mice (BALF).	NF-κB
**Sagiv et al., 2018** [76]	Elucidate the function of airway epithelial p53 in chronic lung inflammation	Mice C57BL/6J female or CCSP-Cre, or CCSP-p53^−/−^ >> exposed to PBS alone or PBS + LPS for 30 min, 3 times a week * 10 weeks >> mice harvested 48 h after last exposure Acute exposure >> LPS, once for 24 h >> harvest **Groups:** CCSP-Cre and CCSP-p53^−/−^ mice. (Mice expressing Cre recombinase from the club cell secretory protein (CCSP) promoter (CCSP Cre mice) crossed with mice in which exons 2 to 10 of p53 genes are surrounded by LoxP sites (P53-loxP or also called CCSP-p53^−/−^)) **Exposure:** Intranasal, 0.5 mg/mL LPS No recovery period.	FACS analysis, RT-qPCR, histological analysis, ELISA, tissue immunostaining	LPS ↑ total alveolar macrophages (AMs) in both p53 (CCSP-Cre) and p53-knockout (CCSP-p53^−/−^) mice. LPS ↑ total BAL AMs in p53 knockout mice compared to p53 mice. LPS ↑ lung interstitial macrophages in both p53 present or knockout mice but ↓ significantly in p63 knockout compared to p53 mice. LPS+ DMSO also ↑ total lung interstitial macrophages.	LPS ↑ total CD3^+^ T cells, CD4^+^ T cells, and CD8^+^ T cells in BAL of both p53 and p53 knockout mice, but they ↓ in p53 knockout compared to p53 mice. Similar results observed in the lungs after BAL was removed. LPS + DMSO ↑ total BAL T-cells (CD3^+^, CD4^+^, and CD8^+^), NK cells, and lung cells (CD3^+^, CD4^+^, and CD8^+^), which were ↓ by Bcl-2 family inhibitor (ABT-737).	LPS ↑ IL-6, CCL2, and KC in BALF of p53 and p53 knockout mice compared to control (PBS). LPS ↑ relative TNF-⍺, IL-6, KC, CXCL2, CXCL5, CCL2, and CXCL10 in lung tissue of both p53 and p53 knockout mice, but their expression ↓ (except IL-6 and KC in lung tissue) in p53 knockout than p53 mice.	p-53 pathway

day or days, d; hour or hours, h; ↑, increase; ↓, decrease.

**Table 2:** Functional response of alveolar macrophages and lymphocytes due to organic dust extract exposure and lipopolysaccharide in mice, cell lines, and human and involved Signaling pathways.

References	Objectives/Hypothesis	Methods: Sensitization or Exposure Sequence	Methods: Assays	Macrophages Receptors; Macrophages Study Conclusion	Lymphocytes Related Findings	Cytokines	Signaling Pathway
**Andelid et al., 2021** [33]	Nature of macrophage-epithelial signaling driving mucus hyperproduction and comparing the LPS and steroid responsiveness of clinically sampled macrophages isolated from sputum plugs and lung resection tissue	Patients with chronic bronchitis (CB) with at least 3 months a year during 2 consecutive years (n = 9). Inclusion visit >> induced sputum and blood samples collected within 3 weeks. For alveolar macrophages (AMs) study, CB, n = 7 and COPD, n = 10. All current or past tobacco smokers Sputum or alveolar macrophages >> 50 nM dexamethasone (DEX) pretreatment >> LPS exposure Sputum or AM cells **exposure**: 100 ng/mL LPS	Oral interview and questionnaires, forced expiratory volume (FEV1) measurement, RNA isolation, RT-PCR, immunoassay, ALI culture, proteome profiler assay—43 human kinases	Higher expression of neutrophils and macrophages, followed by lymphocytes and eosinophils. Conditioned media from LPS stimulated AMs increased MUC5B in human epithelial cells (HBEC) and also increase phosphorylation of kinases CREB and c-Jun on S133 and S63 compared to stimulation with LPS alone. Sputum macrophages were resistant to dexamethasone (steroid) compared to CB/COPD AMs.	N/A	DEX reduced TNF-α in alveolar (75% vs. vehicle, n = 4) and non-alveolar macrophages (72% vs. vehicle, n = 4) on LPS exposure with DEX pretreatment, but not in sputum macrophages (24% vs. vehicle, n = 5). AMs stimulated by LPS showed increase in TNF, IL-1β, and CCL3.	N/A
**Nordgren et al., 2019** [18]	Determine how the lung inflammatory and immune responses to organic dusts are altered by bovine milk-derived dietary extracellular vesicles	Mice >> fed extracellular vesicle-enriched (EV) diet or disrupted extracellular vesicle (DEV) milk containing >> challenge with ODE i.n. or vehicle (saline) >> sacrifice mice after 5 h of final ODE exposure. Acute: ODE exposed once. Repetitive: ODE exposed * 15 consecutive weekdays (3 weeks) Primary peritoneal macrophages isolated from mice settled in plate * 18 h. >> treated with or w/o 1% ODE * 36 h. MH-S cells culture >> treated with 1% ODE, milk EVs, or 1% ODE + milk EVs * 24 h **Exposure** (**mice**)**:** Intranasal, 12.5% ODE **Exposure** (**cells**): 1% ODE	ELISA, histopathological scoring (H& E staining)	In acute exposure, ODE ↑ macrophage counts in DEV diet fed mice. Repetitive ODE exposure ↑ macrophages, total cell influx, and M1 macrophages associated cytokines.	Acute ODE exposure in mice did not affect level of lymphocytes. Repetitive ODE exposure ↑ lymphoid aggregate, alveolar inflammation, and bronchiolar inflammation in the lungs in EV diet fed mice compared to DEV diet fed mice.	Repetitive ODE exposure ↑ IL-6, TNF-⍺, and CXCL1 in mice compared to saline-challenged mice, but ↓ IL-10. 5- or 7-week EV diet + ODE, (peritoneal macrophages): ↑TNF-⍺, IL-12/23, ↓IL-10 and ↓arginase compared to DEV + DE. Murine macrophage (MH-S) + ODE + EV diet: ↑IL-6, TNF-⍺, and IL-12/23, ↓IL-10, and arginase (trend) in cell lysates.	N/A
**Ulu A et al., 2022** [77]	Identify the role of IL-22 in inflammation in response to agricultural dust and impacts of repetitive agricultural dust exposure on macrophage function	Mice >> exposure to 12.5% ODE 5 times a week (50 µL, i.n.) or saline (control) * 3 weeks (total 15 instillations) THP-1 human monocyte cell line culture >> exposed to 1% ODE (24 h) or media control **Exposure** (**mice**)**:** Intranasal, 12.5% ODE **Exposure** (**cells**)**:** 1% ODE	Immunofluorescent staining, scoring of lung pathology, ELISA, qRT-PCR, NanoString gene expression assay	ODE ↑ BALF macrophages in both male and female in IL-22 knockout (KO) mice compared to wildtype (WT) saline control. In female, ODE ↑ BALF macrophages in KO mice compared to KO saline control.	No difference in BALF lymphocytes between saline control and ODE exposed mice irrespective of gender.	ODE exposure ↑ IL-22, IL-22R1 in mice compared to saline control. ODE ↑ total cell counts, neutrophils, polymorphonuclear cells (PMN) in both WT and IL-22 KO mice and macrophages in WT mice.	N/A
**Wang et al., 2018** [78]	*miR-27-3p* controlled TLR2/4 signaling in AMs via targeting the 3′ UTR sequences of PPARγ and inhibiting PPARγ activation	C57BL/6 mice >> cigarette smoke (CS) or LPS or PBS exposure or PPARγ agonist (rosiglitazone) for 3 d Mouse AMs >> transfected with *miR-27-3p* mimics or miRNA scrambles controls, or antisense oligonucleotide inhibitor of *miR-27-3p* or their scrambled oligonucleotides (anti-mRNA control), or incubated with PPARγ agonist GW1929, or CS or LPS for 24 h **Exposure** (**cells**)**:** 300 ng/mL LPS	miRNA analysis, RT-PCR, WB, luciferase reporter assay, immunofluorescence microscopy	AMs treated with CS/LPS upregulated *miRNA-27-3p* (highly upregulated), 1912c, 155-5p, 202, and 883b.	N/A	*miRNA-27-3p* gradually ↓ after 6 h. *miR-27-3p* ↑ INF-γ, IL-1β, TNF-α, and M1 phenotype markers (iNOS and MHCII) and ↓ IL-10 and M2 phenotype markers (CD206 and Arg1). *miR-27-3p* inhibition with CS/LPS exposure ↑ TLR2 and TLR4 in AMs, whereas, deletion of TLR2/4 ↓ proinflammatory cytokines, mRNA expression, and AM polarization. PPARγ agonist before CS/LPS exposure inhibited TLR2, TLR4, and TLR-related genes. PPARγ agonist ↓ INF-γ, IL-1β, TNF-α, and M1 phenotype markers MHCII and iNOS and ↑ in IL-10 and M2 phenotype markers.	TLR2 pathway
**Lee et al., 2017** [79]	Examine the protective effect of the total extract and isolated polyacetylene of D. morbifera leaves in cigarette smoke (CS) and LPS-induced airway inflammation	Mice (C57BL/6N) exposed to different treatments for 1 h >> expose to fresh air or CSE for 1 h for 7 d >> LPS exposed on day 6 >> BALF collection after 24 h Human epithelial tumor cell line, NCI-H292 culture >> after 24 h, incubated with extract, fractions, or isolated compounds [polyacetylene, (9Z, 16S)-16-Hydroxy-9,17-octadecadiene-12,14-diynoic acid] in the presence or absence of CS extract (CSE) for 1 d. (Treatment is mice exposed whole-body to cigarette smoke of 7 cigarettes for 50 min a day for 7 d. **Exposure** (**mice**)**:** Intranasal, 10 µg LPS dissolved in 50 µL of PBS	Cell viability assay, ELISA, hydrogen peroxide (H_2_O_2_) assay, Western blot, H&E staining	CS + LPS ↑ macrophages compared to normal mice. The macrophages level ↓ in ROF (commercial pharmaceutical for COPD treatment) and C10 compared to CSE + LPS exposure.	N/A	↑ TNF-⍺ and IL-6 in CS + LPS, ROF, C10 and T10.	ERK, p38, p65, p-IκB, and NF-κB
**Qiu et al., 2024** [80]	Investigate the anti-inflammatory and anti-airway remodeling effects of eucalyptol, limonene, and pinene enteric capsule (ELP) in a rat model of LPS-induced chronic bronchitis (CB)	Wistar rats >> 50 µL LPS or PBS sprayed on trachea >> ELP or Dexamethasone (DEX) given at different doses except for LPS stimulation days >> on 51st d, rats were fasted for 12 h and sacrificed. (Note: In rats, 50 µL LPS sprayed on trachea on 0 and 7th d, then sprayed 25 µL LPS on 14th, 21st, 28th, 35th, 42nd, and 49th d except in control.) For cell viability, Beas-2B cells culture >> ELP, 1 h >> LPS exposure * 6 h. For immunofluorescence staining, cytokine, and Western blotting, Beas-2B cells >> treated with ELP (various levels) for 1 h >> exposed to LPS for 30 min. >> staining. (Note: ELP: 63, 126, and 252 mg/kg intragastrically once daily or DEX 0.5 mg/kg) (except LPS stimulation days). Control: soybean oil) **Exposure** (**rats**)**:** Sprayed into the trachea, 1 mg/mL LPS	H&E staining, periodic acid-Schiff (PAS) staining, immunohistochemical analysis, transmission electron microscopy (TEM), MTT assay (cell viability assay), RT-qPCR, Western blotting, metabolomic analysis (LC-MS/MS), immunofluorescence staining, and ELISA	LPS ↑ macrophages in rats compared to control and ELP ↓ macrophages compared to LPS exposure.	LPS exposure did not affect level of lymphocytes in BALF in rats.	In rats, LPS ↑ IL-6, TNF-⍺, MIP-1⍺, and CCL5, but ELP pretreatment ↓ them compared to LPS exposed rats. In Beas-2B cells, LPS ↑ IL-6, MIP-2A, CCL5, MCP-1, TNF-⍺ and ELP pretreatment ↓ them compared to LPS exposure.	TLR4, p-ERK, pIRF3, pp65, and p-c-Jun. In BEAS-2B cells: TLR4; p-IKK⍺/β, p-IκB⍺, p-JNK, p-p65, p-p38, p-ERK, p-TBK1, and p-c-Jun.
**Knoell et al., 2019** [81]	Test the effect of zinc in innate immune lung response to ODE and if tissue macrophages intensify the inflammatory process	**Human:** Nutrition survey done on veterans with COPD evaluation of dietary zinc intake (n = 41) **Mice** (**C57/BL6**)**:** zinc-deficient (SZ, zinc: 0.5–1.5 ppm)) or zinc-sufficient (NZ) diet * 3 weeks >> expose to saline or ODE (i.n.). Acute: ODE given once. Chronic: ODE given daily, 5 d a week * 3 weeks **Cell lines:** Murine alveolar macrophage cell line (MH-S) >> cell culture >> pretreated with 10 μM zinc sulphate and 10 μM pyrithione for 30 min >> exposed to 1% ODE Primary human monocytes >> allowed to differentiate with 50 μg/mL GM-CSF for 6 d >> GM-CSF removed >> exposed to 1% ODE **Exposure** (**mice**)**:** Intranasal, 12.5% ODE **Exposure** (**cells**)**:** 1% ODE	ELISA, lung histology (hematoxylin and eosin (H&E) staining), and Western blot	5 h ODE exposure ↑ macrophages and zinc deficiency also ↑ macrophages and total immune cell influx.	N/A	Zinc deficiency ↑ IL-23 and IL-8 in monocyte derived macrophages (MDMs) and IL-23, CXCL1, TNF-⍺, and amphiregulin in MH-S cells compared to control (normal).	PKC catalytic activity (PKCɑ, PKCε, and PKCζ) and NF-κB pathway (p-p65)

day or days, d; hour or hours, h; ↑, increase; ↓, decrease.

## Data Availability

No new data were created were created or analyzed in this study. Data sharing is not applicable to this article.

## Abbreviations

The following abbreviations are used in this manuscript:

ALI	Air liquid interface
AM	Alveolar macrophage
AP-1	Activation protein-1
AREG	Amphiregulin
BALF	Bronchoalveolar lavage fluid
BCA	Bicinchoninic acid
BCR	B cell receptor
CB	Chronic bronchitis
CCL	C-C motif ligand
CCR	C-C motif chemokine receptor
CCSP	Clara cell secretory protein
CD	Cell differentiation
c-Jun	c-Jun N-terminal kinase
COPD	Chronic obstructive pulmonary disease
Cre	Cre recombinase
CREB	cAMP-response element-binding protein
CS	Cigarette smoke
CSE	Cigarette smoke extract
CXCL	Chemokine (C-X-C motif) ligand
CX3CL1	C-X3-C motif chemokine ligand 1
DAMPs	Danger-associated molecular patterns
DEV	Disrupted extracellular vesicle
DEX	Dexamethasone
DHA	Docosahexaenoic acid
ELISA	Enzyme-linked immunosorbent assay
ELPs	Eucalyptol, limonene and pinene enteric capsules
ERK	Extracellular signal-Regulated Kinase
EV	Extracellular vesicle
FEV1	Forced expiratory volume 1
GC	Gas chromatography
H&E	Hematoxylin and eosin
HBEC	Human bronchial epithelial cell
HLA-DR	Human Leukocyte Antigen—DR (“Darren Related”) isotype
HO-1	Heme oxygenase 1
ICAM-1	Intercellular Adhesion Molecule-1
IFN	Interferon
IKK	IκB kinase
IL	Interleukin
ILC3	Type 3 innate lymphoid cells
i.n.	Intranasal
iNOS	Inducible nitric oxide synthase
i.p.	Intraperitoneal
JNK	c-Jun N-terminal kinase
KC	Keratinocyte chemoattractant
KO	Knockout
LPS	Lipopolysaccharide
LTB4	Leukotriene B4
MAPK	Mitogen-Activated Protein Kinase
MaR1	Maresin-1
MEK	Mitogen-Activated Protein Kinase
MCP-1	Monocyte Chemoattractant Protein-1
MHCII	Major Histocompatibility Complex class II
MH-S	Mouse alveolar macrophage cell line
MIP-2	Macrophage inflammatory protein-2
miR/miRNA	Micro ribonucleic acid
MMP-9	Matrix metalloproteases
MPO	Myeloperoxidase
mtDNA	Mitochondrial DNA
MTT assay	3-(4,5-dimethylthiazol-2-yl)-2,5-diphenyltetrazolium bromide assay
MUC	Mucin
MyD88	Myeloid differentiation primary response 88
NADPH	Nicotinamide adenine dinucleotide phosphate
NF-κB	Nuclear factor kappa B
NK cell	Natural killer cell
NLR	NOD-like receptor
NLRP3	NOD-like receptor protein 3
NOD	nuclear oligomerization domain
NOX	NADPH oxidase
NQO1	NADPH dehydrogenase quinone
Nrf2	NF-E2-related factor 2
OD	Organic dust
ODE	Organic dust extract
OVA	Ovalbumin
PAMPs	Pathogen-associated molecular patterns
PARs	Protease-activated receptors
PAS	Periodic acid-Schiff stain
PBS	Phosphate-buffered saline
PGN	Peptidoglycan
PI3K	Phosphatidyl inositol-3 kinase
PKC	Protein kinase C
PLAG	1-palmitoyl-1linoleoyl-3-acetylglycerol
PM	Particulate matter
PMN	Polymorphonuclear cells
PRRs	Pathogen recognition receptors
qPCR	Quantitative polymerase chain reaction
RANTES	Regulated upon Activation, Normal T Cell Expressed and Secreted
RNA	Ribonucleic acid
ROF	Roflumilast
ROS	Reactive oxygen species
RT-PCR	Reverse-transcription polymerase chain reaction
RT-qPCR	Reverse-transcription qualitative polymerase chain reaction
Rx	Treatment
SRA	Scavenger Receptor A
TBK1	TANK-binding kinase 1 (TANK, Tumor necrosis factor receptor-associated factor (TRAF) family member-associated NF-κB activator-binding kinase 1
TEM	Transmission electron microscopy
TH cell	T-helper cell
THP1	Tohoku Hospital Pediatrics-1 human monocyte cell line
TIMP	Tissue Inhibitor of Metalloproteinases
TLR	Toll-like receptor
TNF-α	Tumor necrosis factor-α
UPLC-MS	Ultra-performance liquid chromatography-mass spectrometry/mass spectrometry
WB	Western blot
WT	Wild type
